# A Summary of Current Findings on Quality of Life Domains and a Proposal for Their Inclusion in Clinical Interventions

**DOI:** 10.3389/fpsyg.2021.747435

**Published:** 2021-10-29

**Authors:** Patrick Jones, Peter D. Drummond

**Affiliations:** College of Science, Health, Engineering and Education (SHEE), Murdoch University, Perth, WA, Australia

**Keywords:** quality of life, well-being, intervention, assessment, case formulation, positive psychology, therapy, counselling

## Abstract

Whilst the assessment of quality of life (QoL) and well-being has burgeoned in the past 50 years, there still remains relatively little research into its treatment in psychology, in spite of the launching of such approaches as positive psychology to widen the ambit of interventions to promote well-being. We posit that there are a number of outstanding QoL areas that could be integrated into standard therapeutic procedures, and that this would this result in an increase in well-being as a therapeutic outcome. To investigate this an exploratory search of the literature was undertaken of associations between improvements in a life domain and increased well-being or QoL. Ten domains (relationships, work, money, health, and leisure, mindfulness, self-esteem, resolution of past life events, mental style and life management skills) were identified. In view of the substantial evidence of the cumulative impact of these domains upon well-being, it is proposed that conducting a unidimensional clinical intervention that focuses only on the presenting issue is not sufficient. Implications and possible therapeutic pathways are discussed and it is recommended that practitioners include such QoL domains in their assessment, case formulation, and intervention planning.

## Introduction

The range of evidence-based clinical interventions has certainly grown over recent decades and has gained both popularity and public credibility (Barlow et al., 2016). One approach, however, that has received comparatively less attention is the investigation of domains that are both therapeutically amenable to change and correlate with perceived quality of life (QoL).

Whilst there is now a sizeable amount of research into the predictors of QoL there remains a lack of communication between researchers of QoL and clinicians to assist in the integration of relevant findings, so much so this has even spawned review articles entitled “Findings all psychologists should know from the new science on subjective well-being (SWB)” (Diener et al., 2017) to try to address this dilemma. This lack of cross-pollination has led to research vital to well-being being left undiscovered in clinical fields.

### Objectives

In response to this, we raise the question as to whether there are a number of outstanding QoL areas that could be integrated into standard therapeutic procedures to improve intervention outcomes. And if so we ask what some of the possible approaches could be that may be taken for this to be achieved. As such the objective of this study is to both investigate QoL domains relevant to clinical interventions and, if applicable to identify feasible avenues for their inclusion by clinicians seeking to improve intervention efficacy. The above objectives take up Veenhoven’s (1991) early and largely unheeded call that research that dealt with some of the principles or practices of happiness would provide a good foundation for the development of QoL interventions.

It is postulated that the addition of such domains as part of a therapeutic intervention, will result in an overall increase in both perceived and self-reported well-being, a stated goal of therapy. Such a view is founded on the notion that QoL is a multi-factorial phenomenon, one impacted by a broad range of both objective and subjective influences. Hence it seems reasonable to hypothesize that including more of the domains that make up the overall experience of well-being, will result in an overall increased happiness quotient, as opposed to primarily targeting the presenting issue.

### Conceptualizing Quality of Life

In the early research into QoL, major life areas such as relationships, work, money, health and leisure were seen to play a critical role and hence received much attention (Andrews and Withey, 1976; Campbell et al., 1976; Diener, 1984; Argyle, 1987; Veenhoven, 1991; Diener et al., 1999). For example, in seminal research by Campbell et al. (1976) involving 2,164 respondents, life satisfaction correlated with the following domains: Family Life (0.41); Marriage (0.36); Financial Situation (0.33); Housing (0.30); Job (0.27); Friendship (0.26); Health (0.22); and Leisure (0.21). However, the authors found that the major domains of life satisfaction still only accounted for 15% of the variance in happiness.

Similarly, Andrews and Withey (1976) found that whilst age, sex and occupation correlated with well-being, these variables accounted for only 8% of the variance. Likewise, Kammann (1983) reported that most objective life experiences accounted for no more than a staggering 5% of the variance in measures of well-being. Furthermore, he found that the combination of dozens of life satisfaction domains accounted for fewer than 10% of the variance, leaving him to assert that “objective” life circumstances had a counter-intuitively small impact because they were influenced by multiple “subjective” cognitive processes. It is possibly the complexity of this interaction that has made it hard to integrate into standardized interventions. As such we often find a polarization between goal setting programs (Locke and Latham, 2013) and cognitively based interventions (Hawthorne et al., 2019) rather than a multi-dimensional intervention.

Again, in early seminal research Easterlin (1974, 1995) reported that Americans in the post war decades did not demonstrate an increase in happiness even though income per head had doubled, leading to the “Easterlin paradox” which asserts that circumstances will influence well-being for a limited time before moving back to a set point. Myers (2000) later review over a five-decade span post World War II again found that income in real terms (accounting for inflation) had risen dramatically over this period, yet well-being had not shifted significantly. These findings were in line with Biswas-Diener and Diener’s (2001) research that income had a controversial but generally weak association with happiness, with average estimates being not more than 2–5% of SWB.

Argyle (1999) found that competencies such as intelligence and physical attractiveness had very weak positive correlations with happiness. This accorded with Lyubomirsky (2001) who also found that objective circumstances, demographic variables and life events correlated minimally with SWB. Argyle also found that age had a small positive effect on some aspects of happiness but suggested that this was due to a declining goal-achievement gap. However, this contrasted with later studies that found either no substantial difference in well-being across age (Siedlecki et al., 2013) or U-shaped age profiles in positive hedonic well-being after middle age and a decline in negative hedonic well-being variables such as stress and anger from the early 20s (Blanchflower and Oswald, 2008; Stone et al., 2010; Muhli and Svensson, 2017).

Again, early studies such as that of Brickman et al. (1978) on the transient psychological impact of lottery winning and paraplegia represented *sine qua non-examples* of the failure of objective variables to predict shifts in well-being. This phenomenon was replicated in terms of health which correlated minimally with well-being (Okun and George, 1984). To highlight again the complexity of the relationship between subjective and objective factors, research on the “perception” of one’s general health status was found to correlate more strongly with QoL ratings than “actual” health (George and Landerman, 1984; Argyle, 1987). This is another example of how research findings may lead clinicians to be ambivalent about what they should focus on.

There were attempts, however, to integrate this and explain why such a broad range of life areas, both individually and collectively, were so poorly correlated with the perception of QoL. For example, the Hedonic Adaptation Prevention model was proposed which suggests that people adapt to positive life changes due to the progressive decline in the number of positive events and emotions associated with the once new change. This is coupled with an increase in people’s aspirations for more once they have achieved a goal, which leads to a dissatisfaction with their current level and a gradual return to their well-being baseline (Armenta et al., 2014).

Cummins (2003, 2016) has subsequently suggested that the interaction of these variables occurs within a psycho-social system that maintains well-being homeostasis within a narrow range. He suggested that internal buffers such as self-esteem, optimism, and belief in perceived control along with external buffers made up of resources such as receiving personal assistance, ameliorate the impact of negative events. Cummins acknowledged that poor objective conditions could defeat homeostasis, however, there was typically an expected covariation between objective and subjective indicators.

Over time in response to the poor predictive performance of objective factors there was a shift toward the subjective factors that might play a role in predicting QoL ratings (Sirgy et al., 2006). As the research evolved, along with both straddling the issues of measurement (Kimberlin and Winterstein, 2008), and beginning to include the concepts of well-being and life satisfaction, subjective factors began to take on a far larger role, highlighting that the perception of QoL was indeed a multi-factorial construct. These included the dominant role of affect (Lykken and Tellegen, 1996; Carter, 2004) and the diverse range of cognitive processes implicated in the assessment of one’s well-being (Lyubomirsky, 2001; Layous and Lyubomirsky, 2013).

In an attempt to capture the construct more fully, Diener et al. (1999) carried out an extensive three-decade review of the QoL research and found further evidence to support the multifactorial quality of happiness. In a somewhat reductionist distillation of findings, they found that happy people were most likely to be at the top of the social ladder, typically married and got on well with friends and family. They usually were healthy both physically and mentally, were active and open-minded and felt in control of their lives. In addition, their aspirations concerned social and moral matters rather than money-making, and they frequently were on the conservative side of the political divide.

In terms of objective domains, Diener et al. (1999) in their review identified six domain satisfactions: work, leisure, health, finances, family and one’s group. In a subsequent review, Sin and Lyubomirsky (2009), combined results from 51 randomized controlled interventions and found, in terms of subjective factors, that people engaging in a positive mental style such as thinking gratefully or optimistically or demonstrating increased mindfulness, became significantly happier (Layous and Lyubomirsky, 2013).

In addition, people’s well-being was also found to receive a boost from a range of other factors like positive life events (Kahneman et al., 1999; Lucas et al., 2003), stable positive self-concept (Huebner et al., 1999; Hutz et al., 2014), or the ability to set goals and be self-directing (Deci and Ryan, 2000; Steca et al., 2015; Threadgill and Gable, 2018). With the added inclusion of subjective factors along with the objective life domains (how I feel about my life circumstances), much more of the variance in well-being was explained (Vella-Brodrick et al., 2008; Geerling and Diener, 2018).

### Previous Efforts to Integrate Happiness Research and Therapy

When we look at the early research, we find the call to integrate QoL research into standard therapeutic procedures has been ever present. For example, seminal researchers like Fordyce (1977) lamented that “There is, however, one very important area of happiness research that has not yet received attention—attempts to increase personal happiness” (p. 510). In response to this he developed one of the first packaged happiness enhancement training programs known as the Personal Happiness Enhancement Program (PHEP). Based on the happiness literature Fordyce (1983) subsequently identified what he saw as fundamental cognitions and behaviors which he operationalized into 14 fundamental strategies.

Fordyce (1983) tested his program with 64 both male and female students (mean age = 24.5 years). He reported that “81% of subjects claimed actual increases in their happiness as a result of their learnings” (p. 489). Smith et al. (1995) added to this program by investigating the effect of combining the PHEP with meditation training. They found that the meditation plus PHEP group significantly improved on all dependent measures over the control group and the PHEP group.

One would have thought that such early and promising beginnings would have heralded a new age in clinically oriented QoL interventions. However, most QoL researchers maintained their focus on predictors and assessment, whilst the majority of clinical interventions research continued to favor pathology and symptom reduction over QoL and well-being promotion.

There have, however, been some exceptions and approaches that have aimed to bring QoL research into clinical settings. For example, Seligman and Csíkszentmihályi’s (2000) launching of positive psychology aimed to widen the ambit of clinical interventions to promote well-being and focused on three components: positive institutions, positive experiences and emotions, and positive attributes. Over the years positive psychology has become a credible based-evidence field focused on how to assist people to improve their lives and flourish. It’s focus is on developing practical tools and techniques to improve peoples’ lives.

Peterson and Seligman (2004) subsequently produced the Values-in-Action Classification of Character Strengths which identified 24 character strengths that together produced six higher order virtues: wisdom, courage, humanity, justice, temperance, and transcendence (Ruch et al., 2019). Similarly, around this time, Frisch (1994, 2016) developed QoL therapy, where participants are taught skills to set goals in sixteen areas of life correlated with well-being. Participants are encouraged to either: change their circumstances, attitude, goals and standards, priorities, or boost their satisfaction in other areas.

Over the years several models developed that can be divided into hedonic and eudaimonic conceptualizations of well-being (Hanley et al., 2014). The former, typically referred to as SWB, defines well-being more in terms of the presence of positive affect, absence of negative affect (Kahneman et al., 1999), and the cognitive appraisal of life satisfaction (Diener et al., 1985). By contrast the eudaimonic approach, often referred to as psychological well-being or happiness, focuses more on psychological functioning, human potential and meaning (Ryff and Keyes, 1995; Peterson and Seligman, 2004).

In some research this division has been reconceptualized as well-being processes (eudaimonic processes of engagement) and well-being outcomes (hedonic affects such as life satisfaction and affect) (Bhullar et al., 2013). However, what is shared in these diverse conceptualizations is an agreement that well-being is a multi-factorial construct (Michalos, 2017), hence targeting it with unidimensional interventions could be naïve and even unscientific.

The above observations indicate that there have been sporadic and yet convincing attempts to bring more of a health focus to traditionally pathology focused approaches, yet the latter remains the dominant paradigm. We argue, in view of the insufficient dialogue, that it is imperative to make headway in building the relationship between psychological interventions and QoL research. To bring to the surface more well-being data relevant to therapy, we will briefly summarize some of the findings from 10 QoL domains and discuss the well-being implications for clinical interventions.

## Methods

In response to the research question that asked what influential QoL areas could be integrated into therapy, an exploratory search was conducted of empirical studies that reported significant correlations between a life domain and well-being or perceived QoL. Psychology databases PsycINFO and PsycARTICLES were searched with the keywords of “quality of life,” “subjective well-being,” and “empirical studies,” between 1985 and 2017 to include foundational literature and more recent contributions that built upon these foundations. Search results were divided into so-called objective life areas (such as money or health) and subjective components (mental style topics such as self-esteem or mindfulness).

As the rationale of the inquiry was to show evidence of the impact of a range of life domains upon QoL, and to then argue for their inclusion in therapy and group programs, the number of areas chosen was limited to a clinically manageable 10 areas (five objective and five subjective life domains). A limited number of life areas is seen as manageable within planned clinical interventions (Sakiris and Berle, 2019), and was in line with recommendations from Barlow et al. (2016) who propose “the possibility of distilling a set of psychological procedures that would comprise a unified intervention” (p. 838).

The selection criteria for the final 10 areas were based upon weighing up frequently occurring domains in the literature with the areas reported as having the most impact upon well-being or perceived QoL. For the objective domains there was sufficient evidence that the following areas had a significant correlation with well-being and were worthy of further consideration: relationships, work, money, health and leisure. Similarly for the more subjective domains, mindfulness, self-esteem, resolution of past life events, mental style, and life management (administration) were chosen for investigation. A minimum of 10 studies per domain was seen as a sufficient number to cover different dimensions of each domain.

## Summary of Current Findings on 10 Quality of Life Areas

The following section briefly summarizes the findings from the QoL areas that were identified in the exploratory search of empirical studies that reported significant correlations between life domains and well-being (for more detail on the specific facets see Supplementary Appendix).

### Relationships

Seen as pivotal in the experience of well-being, relationships are the most significant of all the life conditions (Argyle, 1987; Headey and Wearing, 1988; Inglehart, 1990; Demir and Weitekamp, 2007; Proulx et al., 2007; Carr et al., 2014). Multi-axial in nature, relationships can include intimate relationships, family, friends, and colleagues, with satisfaction mediated by the perception of connection, bonding and nourishing interaction with others (Lucas and Dyrenforth, 2006; Gere and Schimmack, 2011).

Close bonds may generate both positive and negative effects on well-being. A case in point is Warr and Payne’s (1982) early study that found that for 1,964 men and 1,113 women in Britain, the family emerged as a major source of pleasure and strain. For women (41%), particularly housewives (56%) the family was a source of strain, although conversely it was identified as a source of pleasure by 31% of the men and 37% of the women. Of all relationship states, marriage has been found to have the strongest effect, with the married being on average most happy, the divorced second and separated last (Myers, 1999; Gottman et al., 2017).

Relationships deliver the experience of intimacy and closeness (Reis, 2012), provide a buffer for stress and social support (Brannan et al., 2012), and help to achieve better health outcomes (Taylor, 2010; Tay et al., 2013). Similarly, the more social relationships people have, the more positive their sense of well-being (Lucas et al., 2008; Tan and Tay, 2017). Whilst falling out with others or reporting a lower relationship quality can have a negative effect upon happiness (Williams, 2009; Bookwala, 2014), relationship health has responded well to both cognitive behavioral therapy (CBT) and behavioral marital therapy (Shadish and Baldwin, 2005; Nirmalan, 2014).

### Work

Work is also considered to play a significant role in the perception of QoL (Argyle, 1999; Van Katwyk et al., 2000) and includes the work conditions and psychological processes under which a sense of well-being at work evolves (Cranny et al., 1992). Employee counseling (Ahmad, 2013) and supportive management has been found to affect vocational satisfaction and QoL reports (Monnot and Beehr, 2014), whilst obstructive management and conflict with program mandates (Shier and Graham, 2013) has been linked to lower SWB (Mathieu et al., 2014).

Feeling stimulated and achieving success at work has also been consistently linked to better health and greater life satisfaction (Shimazu et al., 2015). Conscientiousness or the propensity to be goal directed, plan and delay gratification in work settings is positively related to job satisfaction and SWB (Judge et al., 2005; Carter et al., 2015), although some have argued it has a curvilinear relationship to work outcomes (Astakhova, 2014). The satisfaction of an individual’s basic psychological needs at work relates positively both to their performance and personal well-being (Bryson et al., 2017), and having autonomy for decision making and work planning plays a significant role in the maintenance of that well-being.

Whilst a comprehensive research focus for decades has identified many variables associated with work satisfaction it has also drawn criticism at the lack of defined job satisfaction constructs (Büssing and Bissels, 1998; Eschleman and Bowling, 2009). Similarly, it has been criticized for distortion effects such as the unclear partialling out of work satisfaction from other independent dimensions such as social desirability, cognitive dissonance or attitudinal variables (Unanue et al., 2017).

### Money

Money also appears to play a small role in the perception of QoL, albeit a limited but complex one. Haggerty and Veenhoven’s (2003) longitudinal study of the relationship between life satisfaction and gross domestic product/person across 21 nations revealed that increasing national income correlates with increased national happiness. Whilst the findings were mixed (Blanchflower and Oswald, 2004; Davies, 2010), the effects were generally positive (Stevenson and Wolfers, 2008).

Diener and Biswas-Diener (2009) found that whilst there are strong correlations between national wealth and reports of SWB, there are mostly small correlations between income and well-being within those nations. Similarly, whilst economic growth is accompanied by only a small rise in SWB (Böckerman et al., 2015), increases in individual income can lead to increased well-being when it means avoiding poverty or allows the individual to fulfill material desires in keeping with their values (Srivastava et al., 2001).

However, whilst money can be a robust indicator of SWB (Dolan and White, 2007), if material goals are prized more than other values people appear to be less happy (Diener and Biswas-Diener, 2009). By contrast, financial problems are a strong predictor of clinically diagnosed depression (Huppert and So, 2011), and how people use their income (e.g., spending income on others’ predicted happiness) also impacts on well-being (Dunn et al., 2008). In response to this, financial counseling and goal setting have been found to have positive outcomes on a range of measures including financial capability, health and well-being (Brackertz, 2013).

### Health

The burgeoning area of health-related QoL research highlights how highly health is held in terms of its impact upon QoL (Cummins et al., 2004; Salvador-Carulla et al., 2014; Brazier and Tsuchiya, 2015). Lee et al. (2013) highlight a reciprocal relationship between health status and QoL by arguing for the relevance of well-being for resource allocation decisions within health and social care. For example, positive and negative emotions have been found to correlate with symptom checklists (Krampen, 1999), along with other health-related QoL indicators such as asthma (Christie et al., 1991), physical functioning, pain and general health (Jenkinson et al., 1993; Karimi and Brazier, 2016).

The relationship between health and SWB also appears to be bidirectional. For example, people with illnesses such as heart disease, arthritis, and lung disease often present with increased levels of depressed mood and impaired hedonic and eudemonic well-being (Boehm and Kubzansky, 2012). Conversely, Steptoe et al. (2015) analysis of a longitudinal study identified that reduced well-being was associated with decreased survival (29.3% of people in the lowest well-being quartile died during the 8 year follow-up period compared with only 9.3% of those in the highest quartile).

In terms of interventions, this two-way relationship can be influenced by either regular physical activity to increase well-being (Windle et al., 2010), or actions taken to protect well-being by reducing cortisol output during the day (Steptoe et al., 2012). Personal training interventions that incorporate both aerobic and anaerobic exercise (Patel et al., 2017) improve health-related QoL and cognitive function (Sjøgaard et al., 2016).

### Leisure

The research on leisure has identified a small but significant positive association with mental health, personal needs gratification (Tinsley and Eldredge, 1995; Lloyd and Auld, 2002; Tinsley et al., 2002) and life satisfaction (Rodríguez et al., 2007). Identified as a multidimensional construct that encompasses both structural and subjective aspects, the presence of either discretionary time free from obligations (Kelly and Godbey, 1992; Mitas, 2010), recreational activity under voluntary control (Argyle, 1999) and the pursuit of rewarding activities that transcend daily concerns (Larson et al., 1986) all promote well-being (Sonnentag and Niessen, 2008).

Newman et al. (2014) found five core psychological mechanisms of leisure linked to SWB: detachment-recovery, autonomy, mastery, meaning, and affiliation. Advocating a bottom-up theory of well-being, they posited that the presence of these components leads to an enhanced global experience of well-being. Similarly, Csíkszentmihályi (1990, 2003) suggests in his theory of flow that leisure could also offer peak states of well-being if it included complete concentration, effortlessness and ease and the sense of leisure being intrinsically rewarding (Csíkszentmihályi, 2014). As it can act as a buffer of stress, educating people about leisure has been found to facilitate a sense of resilience that can play a protective role in the maintenance of well-being (Denovan and Macaskill, 2016).

### Mindfulness

In respect to the subjective domains identified as mediating well-being, an individual’s ability to have sufficient control over their cognitive or affective processes (Ingram et al., 1998; Segal et al., 2002; Diener, 2017) is critical. As such, researchers have found that an individual’s capacity for self-examination (Carver and Scheier, 2011) and self-regulation (Linehan, 1993, 2000), are components of mindfulness which may influence well-being (Kabat-Zinn, 1990, 2011; Tacon et al., 2004; Cahn et al., 2013).

Modern mindfulness includes a group of methods that teach individuals to develop non-judgmental awareness that is conducive not only to insight and analysis but also to well-being (Williams et al., 2000). Mindfulness practitioners report a host of benefits including heightened awareness of consciousness or meta-awareness (Schooler et al., 2011), and an improvement in cognitive processing (Lutz et al., 2008) such as improved attentional regulation (Andrews-Hanna et al., 2010; Vago and Nakamura, 2011), executive monitoring (Jha et al., 2007), orienting ability and improved alerting-related processes (Ganaden and Smith, 2011), and better working memory (Brewer et al., 2011). These outcomes are in turn associated with increased QoL (Vibe et al., 2017).

### Self-Esteem

Similarly, the well-researched area of self-esteem presents much evidence that persistent and stable positive self-concept plays a role in the maintenance of well-being (Heatherton and Polivy, 1991; Wong, 2010; Hutz et al., 2014) and life satisfaction (Kwan et al., 1997; Moksnes and Espnes, 2013). Whilst there appears to be a global concept of self-esteem, there is evidence that it is constructed from multiple components which are both internal (e.g., sense of inherent self-worth) and external (e.g., physical appearance, intelligence, social status, level of success) (Marsh, 1990; Harter, 1999; Huebner et al., 1999).

Self-esteem also has a bidirectional relationship with well-being and is strongly and inversely associated with state depression and state anxiety, lending further support to its role in relation to emotional health and well-being (Huppert and So, 2011; Freire and Ferreira, 2018). It is not only responsive to intervention but, in being found to play a mediating role in mental well-being (Kong et al., 2013), has prompted clinicians to develop interventions that specifically address and target self-esteem in the attainment of well-being (Bajaj et al., 2016).

### Life Events

The effects of life events upon well-being have received less focus than the more traditionally associated areas such as self-esteem, work or relationships (Klumb and Baltes, 2004). In earlier research Suh et al. (1996) suggested that “the potential influence of events on positive outcomes, such as SWB, remains relatively unexplored” (p. 1091). This deficit could be due to the methodological challenges of reliably mapping this independent of other variables.

In more recent research Clark and Georgellis (2013) found that life events such as marriage, divorce and widowhood had no long-term effect (contrary to early research by Headey et al., 1984, who found death of a spouse to be significant 2 years later), whereas unemployment still had a negative impact on SWB after 5 years. Margolis and Myrskylä (2011) found that happiness increased around the birth of the first child but, in line with set point theory which suggests that well-being is offset positively within a small range (Cummins et al., 2014), it subsequently returned to the pre-birth level.

There also appears to be gender differences in processing challenging or traumatic life events. For example, Lamoureux-Lamarche and Vasiliadis (2017) found that exposure to violence, an accident or sexual trauma were more likely to be associated with post-traumatic stress syndrome in women than men. Conversely, a life-threatening disease was associated with a reduced life satisfaction only in men whereas life-threatening disease of a close one was significant for women. However, like most subjective variables, the effect was ameliorated by the presence of some kind of intervention or support (Pocnet et al., 2016).

Part of the complexity and hence comparative scarcity of research on the impact of life events is that cross-sectional designs are limited by the measurement of people to a single point in time and hence can’t account for the impact of adaptation. Furthermore, as its mediated by multiple variables such as the temporal distance between events (Bar-Anan et al., 2006), cognitive style, level of affect and personality, knowledge of the status of objective life conditions in itself is not sufficient to predict levels of well-being.

Similarly, as past events can be used as a standard of comparison to judge present circumstances (Strack et al., 1985; Pedersen and Schmidt, 2011), recent negative events have been found to affect people’s appraisal of their well-being (Campbell et al., 1976; Lyubomirsky, 2001). One method to address this has been the development of self-report measures that simply measured the number of recorded positive minus negative life events. This method aimed to remove some of the above confounds and long-term SWB has been found to be correlated with these kinds of calculations (Pavot et al., 1991; Diener and Fujita, 1995; Plagnol and Scott, 2011). Further research into the impact of life events upon well-being could benefit from controlling for such variables.

### Mental Style

A range of psychological mechanisms have been found to mediate the effect of life events upon self-report of happiness (Day et al., 2010) such as resilience, coping style, level of affect or capacity for adaptation (Brickman et al., 1978; Luhmann et al., 2012). Cognitive adaptation includes the sets of basic adaptive processes that intervene between stress and its psychological (including the presence of positive affect and negative affect), social and physiological outcomes (Wong and Lim, 2009; Luhmann et al., 2012; Lyubomirsky, 2013).

For example, through the triggering of cognitive and affective regulatory functions that switch on either behavioral activation or inhibition (Higgins et al., 1999; Freund and Baltes, 2002), individuals could choose the best responses to rebound from the unhappiness of an abusive relationship once the relationship ends (Arriaga et al., 2013). This in turn serves a broader evolutionary function (White, 1974) of protecting, maintaining or enhancing well-being.

Sometimes described as the construal theories of happiness (Van Doorn et al., 2012), this area of research explores the multiple cognitive and affective processes utilized in the attainment of well-being (Lyubomirsky, 2001; Layous and Lyubomirsky, 2013). For example, in semantic network theories it is suggested that emotion can serve the function of activating a network of memories that enhances the accessibility of relevant beliefs about the self and specific life experience (Forgas and Bower, 1987; McIntosh, 2000; Forgas, 2012).

A ruminative, self-focused mental style, however, interferes with these problem-solving skills and the ability to generate effective solutions which, in turn, increases depressed mood and compromises well-being (Nolen-Hoeksema and Morrow, 1991; Carver and Scheier, 2015). By contrast, strong self-determination (Ryan and Deci, 2014), positive self-rating in social comparisons (Pavot et al., 1997; Hoorens and Damme, 2012), and a positive interpretation of negative events (Pauls, 2007), are all associated with heightened well-being. Therapeutic modalities that specifically target mental style such as cognitive behavioral therapy and mindfulness are consistently effective in achieving improvements in ongoing well-being including now online interventions (Carlbring et al., 2009).

### Life Management

Finally, the ability to be self-directing, make decisions and set personal goals has traditionally been seen as a sign of psychological maturity (Allport, 1961; Carver and Scheier, 1990; Deci and Ryan, 2000), and indicative of healthy or mature functioning (Ford and Urban, 1963; Strickland, 2016). One’s self-management or successful life administration has often been associated with motivation (Sheldon and Elliot, 1999), and has some role in the perception of QoL and well-being (Prenda and Lachman, 2001; Kleiber et al., 2011; Steca et al., 2015).

Having a clear strategy to achieve one’s life goals is also seen to play a critical part in SWB maintenance (Conrad et al., 2010; Coote and MacLeod, 2012). It has been associated with the reduction in anxiety about one’s life direction (McGrath and Adams, 1999; Coughlan et al., 2016), and focus on life goals has been found to improve the well-being outcomes and treatment engagement of patients with mental illness (Stanhope et al., 2013). In fact training in goal setting to improve perceived progress along the expectation-outcome gap (Michalos, 1985; Levesque et al., 2004) has demonstrated significant results in the augmentation of health and well-being markers, with new gamification technology replicating these results in virtual training (Johnson et al., 2016).

## Discussion

In the exploratory search of studies reporting correlations between improvements in life domains and increased well-being and QoL, we raised the question as to whether there were a number of outstanding areas that warranted inclusion in clinical work. We hypothesized that, in view of the goal of therapy to improve a client’s QoL and well-being, that such an integration of these influential areas, would improve intervention efficacy.

In the investigation we found that there is in fact substantial evidence that each of the reported domains play differing, but mediating roles in the perception of QoL. Considering this it is recommended that they need to be considered by practitioners in the assessment, case formulation and intervention planning stages of their interventions.

### Limitations

Several limitations to the methods chosen in the current investigation have emerged that need to be addressed. For example, the exploratory examination of the literature, whilst serving as a preliminary search to gather information for a more detailed investigation into selected domains, could have had more weight and less selection bias if it had been conducted as a systematic review in line with PRISMA guidelines, included gray literature and two independent reviewers that could have assessed quality more thoroughly (Panic et al., 2013).

Secondly subsequent to the initial exploratory literature search, as there was an interest in integrating the identified domains into a clinical intervention, a decision was made to limit the number for further investigation to 10 areas. In view of the presence in the literature of both objective and subjective life domains (Diener et al., 1999; Rodríguez et al., 2007; Diener and Biswas-Diener, 2009), it was expected that a similar type of division would occur, hence, a decision was made to split evenly the number of areas chosen for examination into objective and subjective domains. On reflection both the constraint of 10 areas and the equality of the division created an unnecessary limitation which may have narrowed the range of identified factors.

Furthermore, the selection criteria for this choice were based upon the 10 most frequently occurring domains with empirical evidence of a significant relationship with SWB or perceived QoL. This selection process could have been more formalized with a standardized approach that included a quality appraisal for internal validity of the individual studies (Forbes et al., 2017).

### Implications

As each of the reported domains have been found to not only have an impact upon well-being but also interact with each other, it seems that to conduct a unidimensional intervention that focuses on one domain such as relationships, or a distressing past life event, does not seem to be sufficient. As there are many other factors that impact an individual’s experience of happiness, we maintain that it would be more clinically responsible to address the larger set of factors that are responsible for the multi-factorial phenomenon of happiness.

There appear to be a few ways to approach this, with one option being the creation of an individual or group interventions that target these areas with the goal to improve QoL and well-being. To practically address the concern that clients typically present with one specific issue that causes them distress, intervention design could include, along with diagnosis-specific treatment, both psycho-education on the impact of these areas upon well-being and achieving a balanced QoL, and exercises to address the indicated cognitive and behavioral repertoires associated with well-being.

That is the delivery of treatment could be integrated into an existing clinical intervention or be a stand-alone QoL goal setting intervention. For example, for the former option, along with regular treatment the client could be trained in the skillsets associated with each of the life domains such as health, relationships and so on. Concurrently with standard cognitive restructuring and behavioral schedules, clients could be given worksheets and guided to set goals, strategies and time-frames across the major QoL domains. Finally, in view of the typical client’s lack of knowledge of these factors, including a psycho-educational component on the identified predictors of QoL (akin to education on the fight-flight response in anxiety interventions), also seems warranted.

Once treatment for the initial presenting issue was complete, the intervention could move to a well-being focus with the client designing and building their own QoL plan. Clients could be taught how to set goals and strategies in each domain with the aim to build a robust foundation for the maintenance of well-being. Counseling could move closer to a coaching or accountability model with ongoing booster or follow up sessions to assist in the maintenance of goals. If new issues emerged, the model could seamlessly integrate standard cognitive-behavioral techniques, before returning to the well-being maintenance model.

Another extension of this research could be the development of a scale constructed from sub-domains of the domains found to have an impact upon well-being. For example, as communication skills are a major component of relationship satisfaction, items could be derived from the components that make up this domain. Such a scale could include items from each of the domains and could be used in conjunction with group programs and individual clinical work to assess pre-post intervention gains.

In view of the critique leveled at unidimensional interventions that they may be inferior to multidimensional approaches further testing of this hypothesis is warranted. To address this, an active control group of a known treatment could be compared to a proposed QoL intervention. Using standardized psychometric well-being measures, pre-post-follow up data could shed more light on the veracity of this claim.

It would also be prudent to test the hypothesis that the proposed ten domains are all relevant for inclusion in therapy by investigating whether any of them have common underlying factors. For example, could the domain of mindfulness and being in the present moment have something in common with the domain of mental style? A factor analysis on the ten suggested areas to identify underlying domains could clarify any overlap. If some domains could be collapsed into each other, this could simplify the intervention.

## Conclusion

An exploration of objective and subjective life domains revealed evidence that they cumulatively impact overall QoL and the perception of well-being. As such it is suggested that they are relevant to intervention design and treatment. It is proposed that unidimensional clinical interventions that miss out these key areas, may be inferior to multidimensional interventions that include them. Further research is required to test this proposition, and it is recommended that both researchers and practicing clinicians consider these domains and their possible impact in the delivery of clinical interventions.

## Data Availability Statement

The original contributions presented in the study are included in the article/Supplementary Material, further inquiries can be directed to the corresponding author/s.

## Author Contributions

PJ contributed to the conception and design of the research and wrote the first draft of the manuscript. PD contributed to manuscript revision, read, and approved the submitted version. Both authors contributed to the article and approved the submitted version.

## Conflict of Interest

The authors declare that the research was conducted in the absence of any commercial or financial relationships that could be construed as a potential conflict of interest.

## Publisher’s Note

All claims expressed in this article are solely those of the authors and do not necessarily represent those of their affiliated organizations, or those of the publisher, the editors and the reviewers. Any product that may be evaluated in this article, or claim that may be made by its manufacturer, is not guaranteed or endorsed by the publisher.
