# Supplementary material for: A Summary of Current Findings on Quality of Life Domains and a Proposal for Their Inclusion in Clinical Interventions
Source: Front Psychol. 2021 Oct 29;12:747435. doi: 10.3389/fpsyg.2021.747435 (PMC8586497; doi:10.3389/fpsyg.2021.747435)
Supplement: Supplementary file 1 [file Data_Sheet_1.docx]

**APPENDIX**

**QOL DOMAIN FACETS**

**QOL 1 MINDFULNESS**

*Dimension Definition*

Disengagement To be able to actively disengage from habitual cognitive routines

Present Moment Awareness A present time, moment by moment awareness/consciousness of all sensation

Acceptance An ability to have acceptance/equanimity of emotional pleasure or pain

3rd Person Observation To observe mental events as passing not as real entities to be engaged in

Response Flexibility/Freshness To respond uniquely/freshly to each new event (non-habitual)

Perceptual/Diagnostic Clarity The ability to identify and view clearly the nature of one's emotional states

Openness The ability to be open to all experiences

Non-reflexive Thought The ability to direct cognitive operations objectively without reference to the self

Deeper Perspective Awareness that what is occurring is in a context of the larger background of life

Inner Reference Point The ability to identify and trust your inner knowing

Interpersonal Equanimity The capacity to treat others non-judgmentally and compassionately

Regulation of Attention The ability to intentionally direct or self-regulate attention wherever required

Thought Suspension The ability to suspend logical thought processes and just be

Relaxation A natural relaxation response to events

Objective Body Awareness Non-critical focus of physical sensation

**QOL 2 GLOBAL SELF-CONCEPT**

*Dimension Definition*

Intellectual Feeling comfortable with one's level of intellectual competence

Social Feeling connected and at ease with other people and their opinions of oneself

Performance / Behaviour Satisfied with one's general behaviour / performance across areas

Attractiveness Accepting of one's appearance and physical abilities / functioning

Self Beliefs Having positive beliefs about the self and one's abilities

Self-Management Ability to realistically monitor thoughts and actions relative to the situation

Intrinsic Motivation The tendency to have one's life goals intrinsically not extrinsically driven

Self-Worth A sense of worth independent of others or performance

Resilience A high level of perseverance

Determinism A sense of potency of action, achievement and control of one's life

Meaning A sense of meaning and purpose

Acknowledgment The degree of self-appraisal dependent upon others

Success / Competition Able to be successful in relation to others

Relationships / Support One's worth in relation to the quality of close relationships

Virtue Living up to one's moral code and sense of virtue

Unconditional Love A sense that one is unconditionally loved by something greater than oneself

Self-Consistency The capacity to be consistent across roles

Authenticity Acting with a full sense of choice and self-expression

**QOL 3 LIFE EVENTS RESOLUTION**

*Dimension Definition*

Vividness of Detail Recall Ability to not focus or dwell on the details of past negative events

Vividness of Affect Recall Ability to not focus or dwell on the negative affect of past negative events

Adaptation/Habituation The ability to disengage from the negative effect of upsetting life events

Interpretation The capacity to interpret past life events in either a neutral or positive way

Initial Response The ability to initially respond to present life events in a neutral or positive way

Contextualising/Contrast To see negative events in the context of other positive events

Sense of Control To feel a sense of personal control about the life event

Resource Management Ability to protect and maintain personal resources during high demand events

Frequency of Dwelling Ability to not frequently dwell on past negative events

Coping Belief Belief that one has the ability to cope with stressful events

Self-Concept Belief that one's sense of worth is independent of life events

**QOL 4 MENTAL STYLE**

*Dimension Definition*

Rumination / Focus Inappropriate levels of time and focus on issues for the means of problem solving

Interpretation To have a realistic not falsely optimistic or pessimistic view on events

Attribution style To not falsely attribute bad events to internal causes and good events to externals

Self-Reference To not self-doubt or rate one's performance/attributes in comparison to others

Locus of Control To feel self-directed and in control of one's life choices

Integrated Perspective The ability to integrate thoughts, emotions, conduct, meaning, others needs etc

Resourcefulness Resourceful management of psychological and environmental stressors

Cognitive Adaptation Flexibility of adjustment to challenging events

Optimism A positive mental style that is opportunity focused and constructive

Positive Self Appraisal Positive beliefs about the self and one's abilities and degree of competence

Positive Daily Functioning Positive approach to the daily events of one's life

Social Self-Worth Ability to successfully interact and be of use to others

Problem Solving Ability to be analytical, strategic, to act, decide, plan and follow through

Support Seeking/Delegation Seeking of help, advice, comfort and support

Disengagement Efforts to disengage or stay away from stressful events

Distraction Engagement in alternative pleasurable activities

Acceptance Accommodating or minimising non-preferred activities

Emotional Regulation Capacity to process and express emotions at the appropriate time and place

Information Seeking Interest in getting information on causes, consequences and strategies of issues

Negotiation Ability to assess priorities and create workable compromises

Force Use of anger and force to change undesirable situations

**QOL 5 LIFE ADMINISTRATION**

*Dimension Definition*

Commitment Determination and willingness to invest effort in tasks and goals

Realistic Goal Setting Capacity to set attainable tasks and projects

Goal Progress The ability to make progress in set tasks

Self-Concordance To set tasks that match implicit values, interests and capacities

Time Management Organising time schedules to complete set tasks

Time Budgeting Making use of time efficiently

Focus / Attention Ability to direct attention and resources to required tasks

Time Perspective Being able to set short, mid and long term goals and projects

Prioritisation The skill to set goal hierarchies or priorities

Organisation Capacity to use systems and structures to achieve goals

Effectiveness To use relevant means to acquire / attain goals

Compensation Maintenance of functioning / goals through the use of alternative means

Future Planning Lifestyle planning for the future rather than living one day at a time

Sense of Control Belief in one's efficacy to control one's life

Support Accessing of support for tasks

Lists Use of "To Do"" lists

Multi-tasking Managing two or more tasks in one time frame

Chunking Breaking up of tasks into manageable units

Role Clarity Having clearly defined work and domestic roles

Assertion Capacity to say no to extra time demands

**QOL 6 RELATIONSHIP**

*Dimension Definition*

Active / Open Listening Ability to openly listen to neutral or challenging material and feed it back

Emotional Expression The capacity to express positive or negative feelings

Conflict Resolution To be able to constructively problem solve conflictual topics

Perspective Taking The ability to see things from another's point of view

Sympathetic Concern The tendency to experience feelings of sympathy and compassion for others

Empathy The ability to feel and understand the personal distress of others

Social Support / Dependability The availability to support others emotionally and tangibly (assistance)

Self-Awareness The ability to be aware of one's emotional and cognitive relationship issues

Rationality The ability to objectively and rationally relate to others in conflict

Role / Boundary Clarity Clear and articulated expectations of roles and boundaries

Work Success Focus Level of focus on competition, power and success over partner focus

Companionship The degree of sharing across important life areas

**QOL 7 WORK**

*Dimension Definition*

Workload Taking on only the work one can effectively manage within a reasonable timeframe

Role Clarity Clarity of role, work demands and outcome expectations.

Work Support The experience of emotional and pragmatic support in work projects

Task Autonomy Latitude for autonomy, flexibility, choice and personal style

Skill Utilisation Opportunity for utilisation of skills and expression of valued abilities

Intrinsic motivation Intrinsically motivated job tasks and work goals

Variety Variation in job content, location, non-repetitive skill and task variety

Work Predictability Clarity of work rules and expected behaviours and outcome measures

Money Appropriate level of pay relative to skill level and peers

Physical Security Safe, environmentally supportive working conditions

Work Relationships Quality of sharing and solidarity versus conflict in work environment

Job Value Personal sense of job importance, contribution, self and others respect

Low competitiveness The lack of personal valuing to outperform others

Mastery The need to master problems and situations

Organisational Commitment Commitment to / satisfaction with the organisational design / process and its success

Low money beliefs Low value attached to financial gains

Coping Style Ability to deal with situations in a non-reactive unemotional way

Work-Related Fatigue The degree of physical, mental or emotional impact of work upon the employee

Job Security The level of doubt versus stability in job security

Task Satisfaction The level of resistance or engagement across work tasks

Value Attainment The extent to which one's job helps to attain personally valued outcomes / goals

Global Work Attitude General feelings and beliefs about the nature of one's job and organisation

Work Mood How people feel at work when they are engaged in their job

Family/Friends Support Feeling supported in one's work by significant others such as family and friends

Work Goals Having short, medium and long term work goals

Personal Life Opportunities Work allows for the obtaining of personal goals

Progression Work offers the opportunity for progression and growth if desired

Perceived control The sense that one's work is under one's personal control

Work Focus The degree to which individuals take their work seriously are active and work hard

**QOL 8 MONEY**

*Dimension Definition*

Absolute Income Absolute not relative size of income

Habituation (-) The degree to which individuals habituate to income gains

Security Interest in money for practical needs, future life planning and freedom from poverty

Family Support View money as a means to offer family support

Market worth See money as a means to get just compensation for efforts

Pride Driven by a sense of pride and achievement

Leisure See money as an opportunity to spend time and resources on leisure activities

Freedom View money as a resource to implement own ideas

Enjoyment Value the availability of money to spontaneously use it how one wants

Charity See money as a resource to assist others

Social Comparison(-) Use money as a means to show off and seek power

Self-Doubt(-) Use money to assist in overcoming self-doubt

Budget Have a written budget that is consistently followed

Future expectations(-) Expectations that one's future income will be better

Savings Consumption is predictably less than expenditure to allow for savings

Minimum Income The difference between actual income and minimum survival income

Debt Tolerance Lack of tolerance for debt accumulation

Impulsivity Tendency to impulse buy and not consistently manage money

Support Support by significant others to stay out of debt and manage money

Work Status Perceived association between the role of work and money management

Budget Attitudes Perception of the utility in maintaining a budget

Income Perceptions Perception that one doesn't make enough money to maintain a budget

Resource Sharing Pooling of money and financial resources of partners in relationship

Consumption Discipline Self-discipline exercised over the urge to spend current income

Income Predictability Income not vulnerable to unforseen life events

Financial Planning Specific planning activities that enhance the probability of achieving financial goals

**QOL 9 HEALTH**

*Dimension Definition*

Physical Functioning Level of disability, mobility, activity and dexterity

Role Limitations Effect of health upon carrying out of life roles

Energy / Vitality Level of daily energy

Health Perception Perception of the status of one's health

Disease Status Presence of diseases

Pain / Discomfort Intensity, frequency and duration of pain / bodily discomfort

Drug side effects Physical effect of drugs

Sleep / Rest Amount of sleep (disturbance) or rest

Mental Health General mood and psychological well-being

Fitness Ability to perform a range of physically demanding tasks

Social Functioning/Support Range of supportive social interactions

Daily Activities Range of daily activities

Mental Alertness Capacity to reason, learn and problem solve

Body care Ability to look after one's own health

Communication Level of communication skills

Future Outlook Positive future outlook

Self-Regulation Sense of personal control

Achievement Achievement of goals that express oneself

Faith Trust in some kind of life meaning

Goal Conflict Conflict between values and behaviours

**QOL 10 LEISURE**

*Dimension Definition*

Exertion Opportunity to carry out the desired level of physical activity

Family Companionship and connection in daily tasks/other activities

Self-Expression Opportunity to creatively express oneself

Challenge Optimal level of fulfilment/satisfaction from life activities

Pleasure The optimal balance of pleasure and sacrifice

Stimulation Sufficient complexity in activities/events to generate stimulation

Achievement Opportunity to achieve something

Down time Undemanding, low effort, time-out opportunity

Domestic Household management activities

Self-Improvement Opportunity for personal growth and improvement

Variety A range of hobbies / interests

Time load Amount of time spent engaging in leisure

Friendships Engagement in activities/topics that transcend mundane daily tasks

Activities Choice of activities such as housework, sport, travelling

Motivation Level of intrinsic motivation determines amount of leisure

Freedom of choice The experience of freedom of choice in the activity

Commitment Commitment to the leisure activity

Concentration Focused concentration on the leisure activity

Forgetting of self Absorption in the activity and forgetting of self

Time loss Loss of time

Flow The experience of engagement when challenges meet skills

Relaxation Calm experience of no stress or tension

Positive affect The experience of happy and satisfied emotions
